# Propofol Induces Cardioprotection Against Ischemia-Reperfusion Injury *via* Suppression of Transient Receptor Potential Vanilloid 4 Channel

**DOI:** 10.3389/fphar.2019.01150

**Published:** 2019-10-04

**Authors:** Binbin Wang, Qiongfeng Wu, Jie Liao, Shaoshao Zhang, Huixia Liu, Cui Yang, Qian Dong, Ning Zhao, Zhengrong Huang, Kefang Guo, Yimei Du

**Affiliations:** ^1^Department of Cardiology, Union Hospital, Tongji Medical College, Huazhong University of Science and Technology, Wuhan, China; ^2^Department of Critical Care Medicine, Zhongnan Hospital of Wuhan University, Wuhan, China; ^3^Department of Cardiology, The First Affiliated Hospital of Xiamen University, School of Medicine, Xiamen University, Xiamen, China; ^4^Department of Anesthesiology, Zhongshan Hospital, Fudan University, Shanghai, China

**Keywords:** propofol, TRPV4, cardioprotection, ischemia-reperfusion injury, calcium

## Abstract

Ca^2+^ entry *via* the transient receptor potential vanilloid 4 (TRPV4) channel contributes to Ca^2+^ overload and triggers many pathophysiological conditions, including myocardial ischemia/reperfusion (I/R) injury. Propofol, a widely used intravenous anesthetic, attenuates myocardial I/R injury. However, the mechanism of propofol remains to be examined. The present study aims to test the hypothesis that propofol attenuates myocardial I/R injury through the suppression of TRPV4. We used a murine *ex vivo* model of myocardial I/R and *in vitro* cultured myocytes subjected to hypoxia/reoxygenation (H/R). Propofol or TRPV4 antagonist, HC-067047, attenuates myocardial I/R injury in isolated hearts. In addition, propofol, HC-067047, or TRPV4-siRNA attenuates H/R-induced intracellular Ca^2+^ concentration ([Ca^2+^]_i_) increase and cell viability reduction. On the contrary, TRPV4 agonist GSK1016790A exacerbates both *ex vivo* and *in vitro* myocardial injury. Pretreatment with propofol reverses the myocardial injury and intracellular Ca^2+^ overload induced by GSK1016790A at least *in vitro*. However, neither the combination of propofol and HC-067047 nor applying propofol to cells transfected with TRPV4-siRNA creates additional protective effects. In addition, propofol dose-dependently inhibits TRPV4-mediated Ca^2+^ entry induced by GSK1016790A and 4α-PDD. Propofol attenuates myocardial I/R injury partially through the suppression of TRPV4 channel and the subsequent inhibition of intracellular Ca^2+^ overload.

## Introduction

Propofol (2, 6-diisopropylphenol), an intravenous anesthetic frequently used during surgery, has been proved to dose-dependently attenuate myocardial ischemia/reperfusion (I/R) and hypoxia/reoxygenation (H/R) injury (Xia et al., 2006; Sun et al., 2017; Zhao et al., 2019). The potential mechanisms include the reduction of reactive oxygen species (ROS) (Xia et al., 2003), the inhibition of Ca^2+^ overload (Kim et al., 2008), the suppression of Ca^2+^ entry channels (L-type Ca^2+^ channel, T-Type Ca^2+^ channel, and Na^+^/Ca^2+^ exchange) (Luk et al., 1995; Kojima et al., 2015), and the reduction of apoptosis (Liu et al., 2017).

An increased level of intracellular Ca^2+^ is detrimental to cardiomyocytes and results in myocardial cell death during I/R injury (Garcia-Dorado et al., 2012). One of our recent studies has shown that Ca^2+^ entry *via* the transient receptor potential vanilloid 4 (TRPV4) channel in cardiomyocytes plays a critical role in mediating Ca^2+^ overload and ROS release during the process of myocardial I/R injury (Wu et al., 2017). Similar to the cardioprotective effects of propofol, TRPV4 blockage reduces infarct size and troponin T (TnT) and improves *in vivo* heart function (Dong et al., 2017). TRPV4 protein levels have also been found to increase in the brain I/R model (Jie et al., 2016), while TRPV4 selective antagonist HC-067047 (Everaerts et al., 2010) attenuates I/R-induced brain injury (Jie et al., 2015). In addition, excessive Ca^2+^ influx through TRPV4 induced by TRPV4 agonists GSK1016790A and 4α-PDD leads to the apoptosis of retinal ganglion cells and neuronal death in the hippocampus (Ryskamp et al., 2011; Jie et al., 2016). Based on these findings, TRPV4 channel is a promising target in the treatment of I/R-induced myocardial injury (Jones et al., 2019; Wu et al., 2019). Moreover, propofol has been found to directly interact with TRP channels, including TRPC5 and TRPA1 (Bahnasi et al., 2008; Ton et al., 2017). However, there is no direct evidence showing that propofol inhibits TRPV4-mediated Ca^2+^ entry. Therefore, in this study, we hypothesize that propofol activates protective mechanisms through the suppression of TRPV4 channel and the subsequent inhibition of intracellular Ca^2+^ overload in *ex vivo* isolated hearts under I/R and *in vitro* cell models under H/R.

We first investigated the dose-dependent protective effects of propofol against reperfusion-induced myocardial injury. We subsequently confirmed the involvement of TRPV4 channel in reperfusion-induced myocardial injury using pharmacological approaches. Furthermore, we observed the effects of propofol on TRPV4 agonist-induced myocardial I/R injury and examined the cardioprotective effects of propofol in TRPV4-siRNA transfected cells. Finally, we analyzed the effects of propofol on TRPV4-mediated Ca^2+^ influx and intracellular Ca^2+^ concentration ([Ca^2+^]_i_) in HEK 293 cells, H9C2 cells, and adult rat ventricle myocytes (ARVMs).

## Materials and Methods

### Animals

Male C57BL/6 mice were purchased from Vital River Laboratories, Beijing, China, and Sprague-Dawley rats were purchased from the Experimental Animal Center, Tongji Medical College, Huazhong University of Science and Technology, Wuhan, China. All animals were kept in the Experimental Animal Center of Tongji Medical College. This study was carried out in accordance with the recommendations of the National Institutes of Health Guide for the Care and Use of Laboratory Animals (NIH Publications No. 8023, revised 1978). The protocol was approved by the the Animal Research Ethics Committee of Tongji Medical College, Huazhong University of Science and Technology. All experiments were conducted on age and body weight-matched animal groups.

### Pharmacologic Agents

The propofol we used on isolated mice hearts (10 mg/ml, Fresenius Kabi GmbH, Graz, Austria) was dissolved in an intralipid suspension including soybean oil (10%), egg phosphatide (1.2%), and glycerol (2.25%). An equivalent volume of intralipid (INTRA, 20%, Sigma Aldrich, St. Louis, MO, USA) was used as a vehicle control. In cell experiments, we applied pure propofol monomers (TCI Shanghai, Shanghai, China), which were dissolved in dimethyl sulfoxide (DMSO). Propofol dosage (25, 50, 100 μM *ex vivo*; 12.5, 25, 50, 100 μM *in vitro*) was based on the volume previous studies applied (Xia et al., 2006; Sun et al., 2017). TRPV4 agonists GSK1016790A (20 nM *ex vivo* and 300 nM *in vitro*) and 4α-PDD (3 μΜ *in vitro*) and TRPV4 antagonist HC-067047 (0.1 μM *ex vivo* and 1 μM *in vitro*) were all from Sigma Aldrich and were dissolved in DMSO. The percentage of DMSO in the final solution was less than 0.01%.

### Langendorff Perfusion and Experimental Protocol

Mice (22–28 g) were anesthetized with 2.5% avertin 0.02 ml/g and heparinized with sodium heparin 125 IU intraperitoneally. Avertin was prepared by mixing 5 g of tribromoethanol (Sigma-Aldrich, St. Louis, MO, USA), 5 ml of tert-amyl alcohol (Sigma-Aldrich, St. Louis, MO, USA), and 195 ml of normal saline solution. After thoracotomy, mice hearts were quickly excised and mounted for retrograde perfusion using a Krebs-Henseleit buffer. The buffer (in mM: NaCl 118, KCl 4.7, NaH_2_PO_4_ 1.2, MgSO_4_ 1.2, NaHCO_3_ 25, glucose 15, CaCl_2_ 2.5, EDTA 0.5, pH = 7.4) was gassed with 95% O_2_ and 5% CO_2_ at 37°C, and its constant flow rate was 2–4 ml/min. A latex water-filled balloon fixed to a pressure transducer was inserted through the mitral valve into the left ventricle to constantly monitor heart rate (HR), left ventricular end diastolic pressure (LVEDP), left ventricular systolic pressure (LVSP), left ventricle developed pressure (LVDP = LVSP-LVEDP), rate pressure product (RPP = LVDP*HR), as well as the maximum increase/decrease rate of left ventricular pressure (± dP/dt max). LVEDP was adjusted to approximately 5 mmHg before the start of the experiment by adjusting the intraventricular balloon volume. Selection criteria include: HR ≥ 300 beats/min, LVDP ≥ 60 mmHg after 15 min of equilibration, maintenance sinus rhythm, and no prolonged arrhythmias (Sutherland et al., 2003).

**Figure 1:**
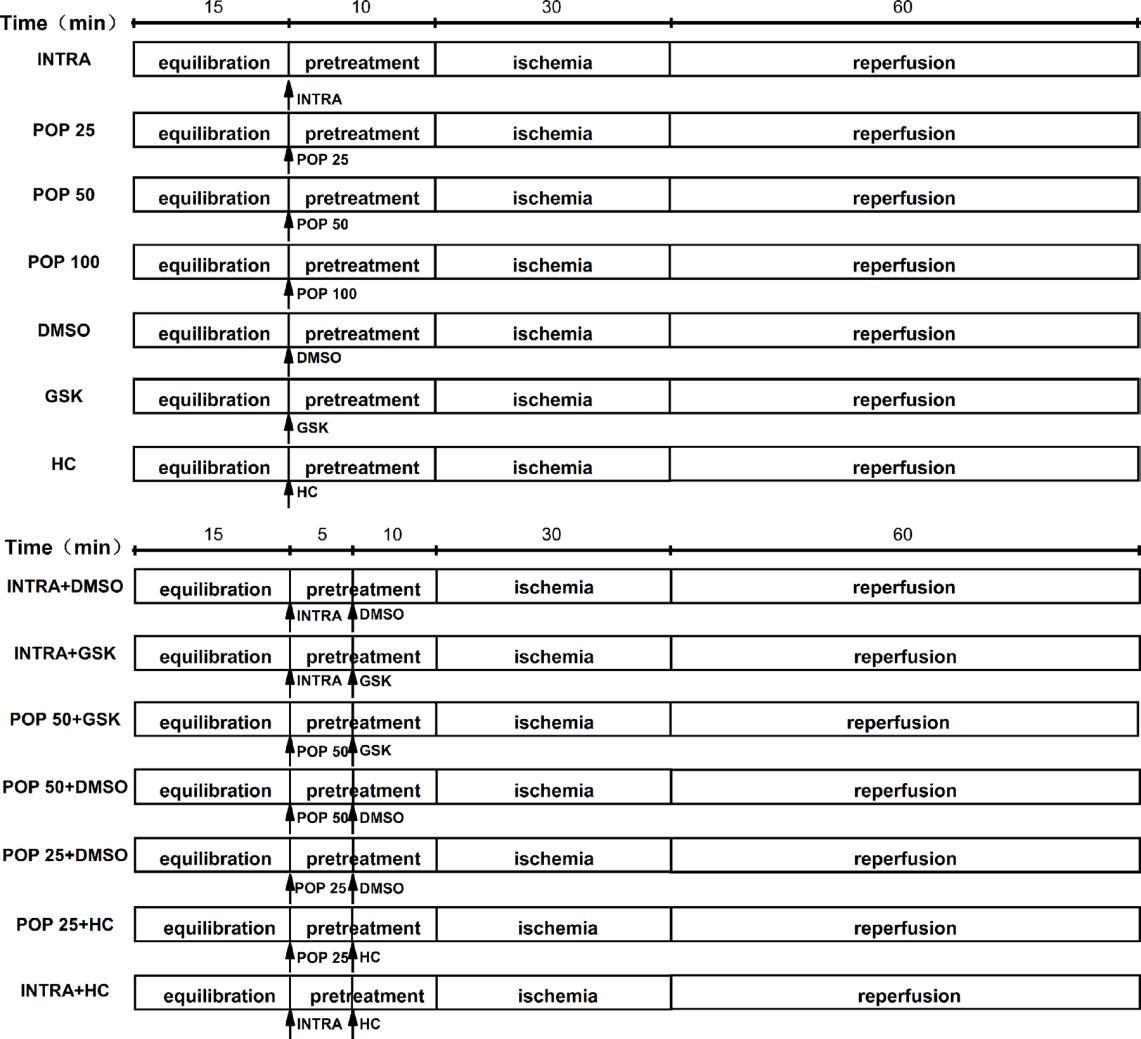
The protocol for myocardial I/R injury in isolated mice hearts.

In the experimental protocol, we divided the mice hearts into 14 treatment groups (**Figure 1**): INTRA, POP 25, POP 50, POP 100, DMSO, GSK, HC, INTRA + DMSO, INTRA + GSK, POP 50 + DMSO, POP 50 + GSK, POP 25 + DMSO, POP 25 + HC, and INTRA + HC. I/R was achieved by 30 min of global ischemia (zero flow) and then 60 min of reperfusion.

### Lactate Dehydrogenase Release

Lactate dehydrogenase (LDH) activity was assessed by an assay kit (Nanjing Jiancheng Biochemistry Co., Nanjing, China) according to the manufacturer’s instructions. Coronary effluent samples were collected 0, 5, 10, 15, 30, 45, and 60 min after reperfusion (Rossello et al., 2016).

### Myocardial Infarct Size

Myocardial infarction size was measured by 2,3,5-triphenyltetrazolium chloride (Sigma Aldrich, St. Louis, MO, USA) staining. At the end of reperfusion, the left ventricle was cut into 1 mm-thick transverse slices from the apex to the base. The size of 2,3,5-triphenyltetrazolium chloride -negative areas (infarct area, white) was calculated using the planimetry function in Image-Pro Plus v 6.0 (Media Cybernetics, MD, USA). The myocardial infarct size is presented as the percentage of infarct area size in the total left ventricle.

### Cell Culture and Treatment

As previously described, adult rat ventricle myocytes (ARVMs) were isolated by enzymatic dissociation from the left ventricles of Sprague-Dawley rats. ARVMs were incubated in a medium comprised of 5% fetal bovine serum (Hangzhou Sijiqing Biological Engineering Materials Co., Ltd., Hangzhou, China), 10 mM 2,3-butanedione monoxime, and M199 (Gibco, Grand Island, NY, USA) at 37°C in 5% CO_2_ for 24 h before experiments. H9C2 cells were purchased from ATCC (Rockefeller, MD, USA) and were cultured in Dulbecco’s modified Eagle’s medium (DMEM, Gibco, Grand Island, NY, USA) supplemented with 15% FBS, 100 U/ml penicillin, and 100 μg/ml streptomycin at 37°C in a humidified atmosphere with 95% air and 5% CO_2_. Cells were treated with TRPV4 agonists GSK1016790A (300 nM), 4α-PDD (3 μM), or TRPV4 antagonist HC-067047 (1 μM) at the onset of hypoxia, while propofol (12.5, 20, 50, 100 μM) was applied 30 min earlier.

H9C2 cells with > 50% sub-confluency were transfected with TRPV4-siRNA (5-GGAGCTGAACAAGAACTCA-3, RIBOBIO, Guangzhou, China) using Lipofectamine 3000 (Invitrogen). A scrambled non-silencing siRNA (5-TTCTCCGAACGTGTCACGTdTdT-3, RIBOBIO) was used as a negative control. The knockdown efficiency of TRPV4-siRNA is shown in the **Supplementary Figure S1**.

HEK-293T cells were purchased from ATCC and maintained in the same conditions as H9C2 cells. HEK-293T cells were transiently transfected with hTRPV4/pCDNA 4.0 (generously provided by Dr. Ke-wei Wang, Peking University School of Pharmaceutical Sciences, Qingdao University School of Pharmacy, China) using the Lipofectamine 3000 method as described previously (Fu et al., 2013).

### H/R Model

Before hypoxia, the culture medium was changed to a fetal bovine serum-free DMEM. Hypoxia was achieved by placing the cells in a controlled hypoxic plastic chamber (HiTech Photelectricity Biotechnology Co., LTD., Guangzhou, China) containing 95% N_2_ and 5% CO_2_ for 6 h. Subsequently, the medium was replaced by a normal medium and the cells were incubated in 95% air and 5% CO_2_ for a 6 h reoxygenation.

### [Ca^2+^]_i_ Measurement

[Ca^2+^]_i_ was measured according to previously described procedures (Wu et al., 2017). Cells were loaded with 2 μM Fluo-4 AM for 30 min. Florescence was captured with the Enspire Multimode Plate Reader (PerkinElmer, Boston, MA, USA). Relative changes in Ca^2+^ influx stimulated by GSK1016790A (300 nM) or 4α-PDD (3 μM) are presented as (F/F0) or fold changes (∆F/F0), respectively. F represents fluorescence intensity, F0 represents the average fluorescence intensity before GSK1016790A or 4α-PDD stimulation, and ΔF represents the mean fluorescence intensity at the steady state after drug stimulation minus F0. Ionomycin (1 μM, Sigma, St. Louis, MO, USA) was set as a positive control.

### Cell Viability Measurement

Cell viability was measured using the Cell Counting Kit-8 (CCK-8, Dojindo Molecular Technologies, Kyushu, Japan) as previously described (Fu et al., 2015; Wu et al., 2017). Optical density at the 450-nm wavelength was measured using a microplate reader (DG5033A, Nanjing, China).

### Statistical Analysis

All values are presented as mean ± SD. Data was analyzed by a two-tailed t test (data on Ca^2+^ influx in H9C2 and ARVMs) or a one-way ANOVA followed by a Bonferroni method analysis (data on *ex vivo* and *in vitro* of I/R and H/R). Statistical analysis was performed using SigmaStat3.5 (Systat Software Inc, San Jose, California, USA). The dose-response curve was fitted using the Hill equation in Origin 9 (OriginLab Corporation, Northampton, MA, USA). IC_50_ represents the propofol dosage that produces 50% of the maximal inhibitory effect. Only when p < 0.05 was the difference considered statistically significant.

## Results

As shown in the **Supplementary Tables S1** and **S2**, all groups showed identical measured values (HR, LVDP, ± dP/dt max, and RPP) after 15 min of equilibration as well as before global ischemia. Original tracings of the left ventricle pressure for each experiment are shown in **Supplementary Figures S2–S5**.

### Propofol Concentration-Dependently Attenuates Myocardial I/R Injury in Isolated Mice Hearts

**Figure 2:**
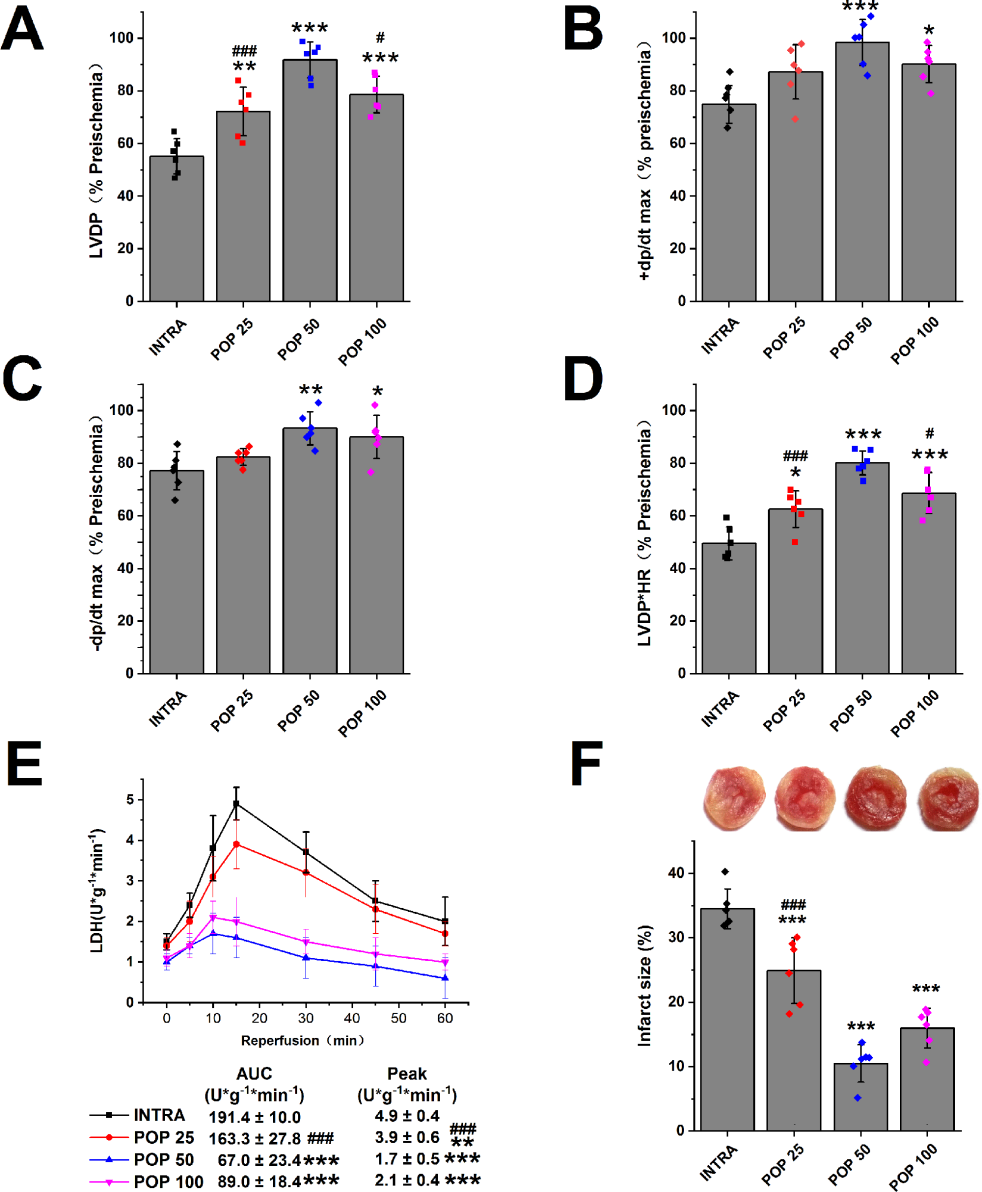
The dose-dependent effect of propofol on myocardial I/R injury. The recovery of LVDP **(A)**, +dP/dt max **(B)**, -dP/dt max **(C)**, and RPP **(D)** 60 minutes after reperfusion displayed as the percentage of respective preischemia values. **(E)** The amount of LDH release in coronary effluent at different times after reperfusion. **(F)** Representative images of TTC stained LV slices. Infarct size is quantified. Values are presented as mean ± SD, n = 6 for all groups, a one-way ANOVA followed by Bonferroni test, *p < 0.05, **p < 0.01, ***p < 0.001 *vs.* INTRA. ^#^p < 0.05, ^###^p < 0.001 *vs.* POP 50.

We first examined the protective effects of propofol against myocardial I/R injury in isolated hearts. **Figures 2 (A–D)** and **Supplementary Figures S2 (A–D)** show that propofol (25, 50, 100 μM) promoted the recovery of LVDP, +dP/dt max, -dP/dt max, and RPP in a concentration-dependent manner. In addition, the hearts pretreated with 50 μM propofol recovered best from INTRA (LVDP increases from 55.1 ± 2.7% to 91.8 ± 2.8%, +dP/dt max from 74.9 ± 2.9% to 98.4 ± 3.6%, -dP/dt max from 77.2 ± 3.0% to 93.3 ± 2.6%, and RPP from 49.6 ± 2.6% to 80.2 ± 1.9%). As shown in **Figure 2E**, propofol reduced the peak value and AUC of LDH release in a dose-dependent manner. Similarly, propofol dose-dependently reduced the infarct size of the left ventricle, and 50 μM propofol had the best protective effects (from 34.5 ± 3.1% to 10.5 ± 2.9%, **Figure 2F**).

### TRPV4 Agonists/Antagonists Aggravate/Attenuate Myocardial I/R Injury in Isolated Mice Hearts

**Figure 3:**
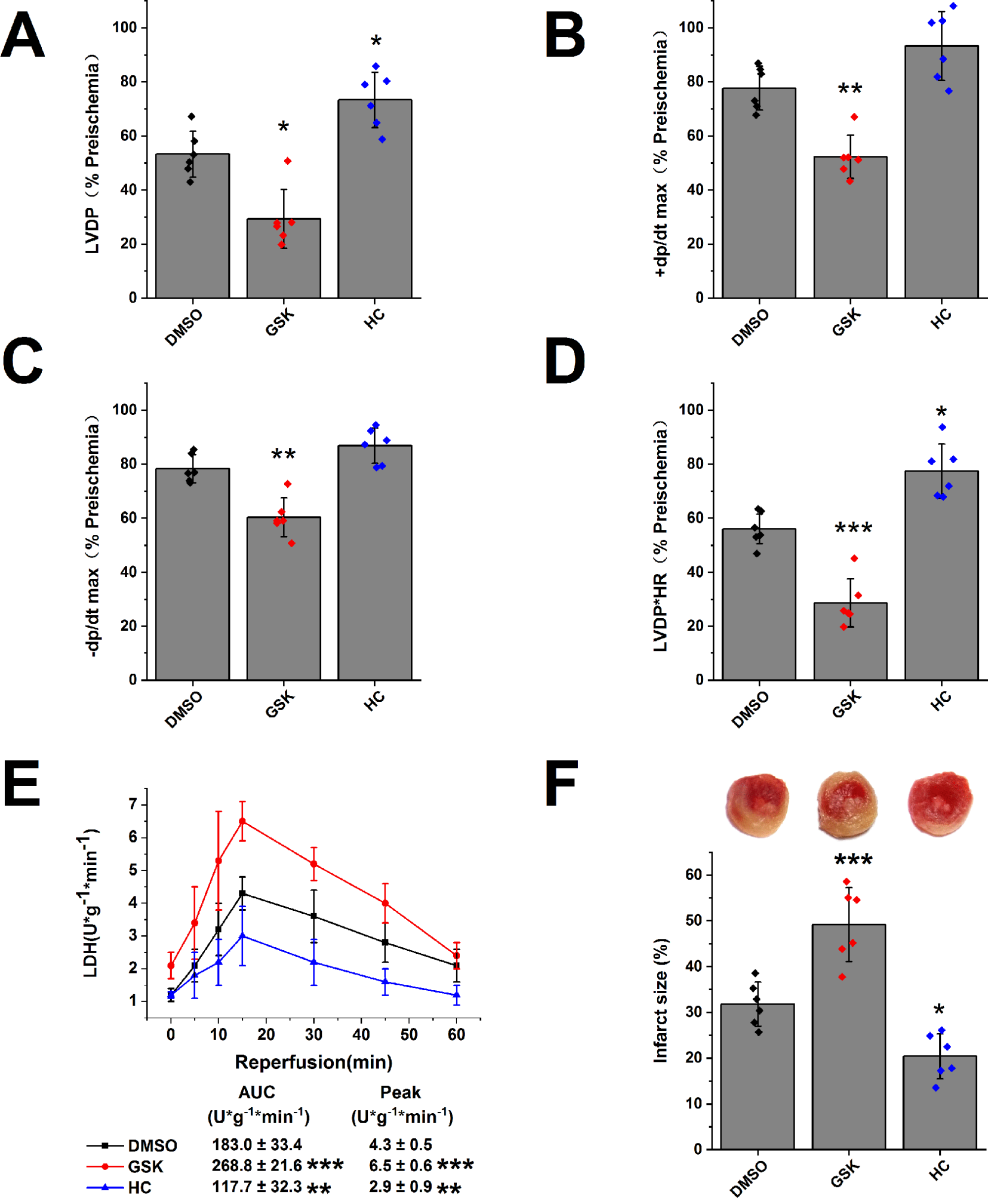
The effect of TRPV4 agonist GSK1016790A and TRPV4 antagonist HC-067047 on myocardial I/R injury. The recovery of LVDP **(A)**, +dP/dt max **(B)**, -dP/dt max **(C)**, and RPP **(D)** 60 minutes after reperfusion displayed as the percentage of respective preischemia values. **(E)** The amount of LDH release in the coronary effluent at different times after reperfusion. **(F)** Representative images of TTC stained LV slices. Infarct size is quantified. Values are presented as mean ± SD, n = 6 for all groups, a one-way ANOVA followed by Bonferroni test, *p < 0.05, **p < 0.01, ***p < 0.001 *vs.* DMSO.

We evaluated the role of TRPV4 channel in isolated hearts after I/R injury. In comparison with DMSO treatment, TRPV4 agonist GSK1016790A impaired the recovery of LVDP, +dP/dt max, -dP/dt max, and RPP (LVDP decreases from 53.3 ± 8.5% to 29.4 ± 10.9%, +dP/dt max from 77.7 ± 8.1% to 52.3 ± 8.0%, -dP/dt max from 78.4 ± 5.2% to 60.4 ± 7.2%, and RPP from 56.1 ± 5.5% to 28.7 ± 8.9%), while TRPV4 antagonist HC-067047 promoted the recovery of the above indexes, as shown in **Figures 3A–D** and **Supplementary Figures S3A–D**. Similarly, TRPV4 antagonist HC-067047 reduced LDH release and infarct size, while TRPV4 agonist GSK1016790A had completely opposite effects (**Figures 3E, F**). Consistent with previous *in vivo* studies (Dong et al., 2017; Wu et al., 2017), these results confirmed that TRPV4 channel plays a pathogenic role in myocardial I/R injury.

### Effects of the Combination of Propofol and TRPV4 Agonists/Antagonists on Myocardial I/R Injury in Isolated Mice Hearts

**Figure 4:**
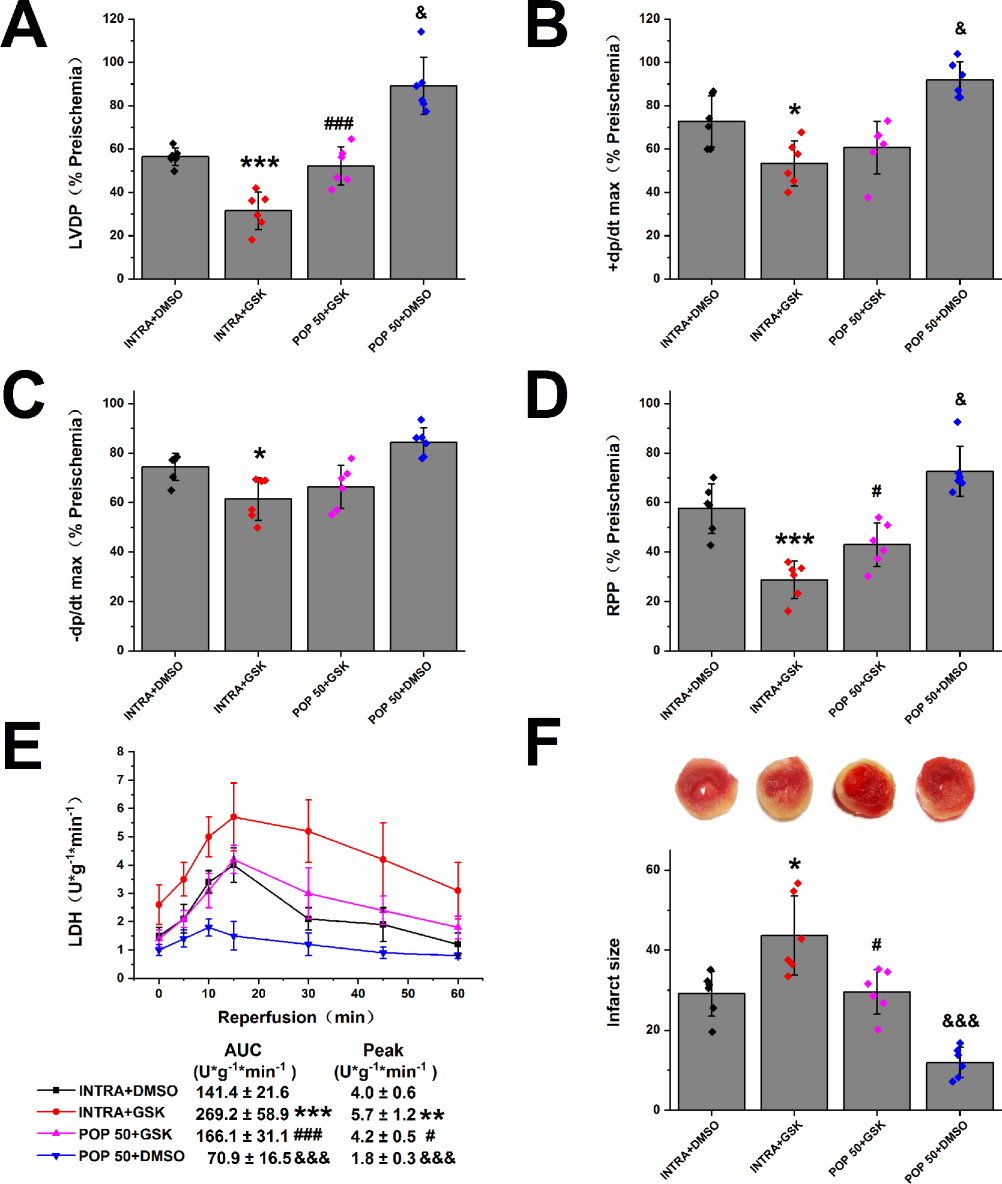
The effect of propofol on TRPV4 agonist-induced myocardial I/R injury. Propofol or intralipid is given 5 minutes before applying TRPV4 agonist or DMSO. The recovery of LVDP **(A)**, +dP/dt max **(B)**, -dP/dt max **(C)**, and RPP **(D)** 60 minutes after reperfusion displayed as the percentage of respective preischemia values. **(E)** The amount of LDH release in the coronary effluent at different times after reperfusion. **(F)** Representative images of TTC stained LV slices. Infarct size is quantified. Values are presented as mean ± SD, n = 6 for all groups, a one-way ANOVA followed by a Bonferroni test, *p < 0.05, **p < 0.01, ***p < 0.001 *vs.* INTRA + DMSO. ^#^p < 0.05, ^###^p < 0.001 *vs.* INTRA + GSK. ^&^p < 0.05, ^&&&^p < 0001 *vs.* POP50 + GSK.

**Figure 5:**
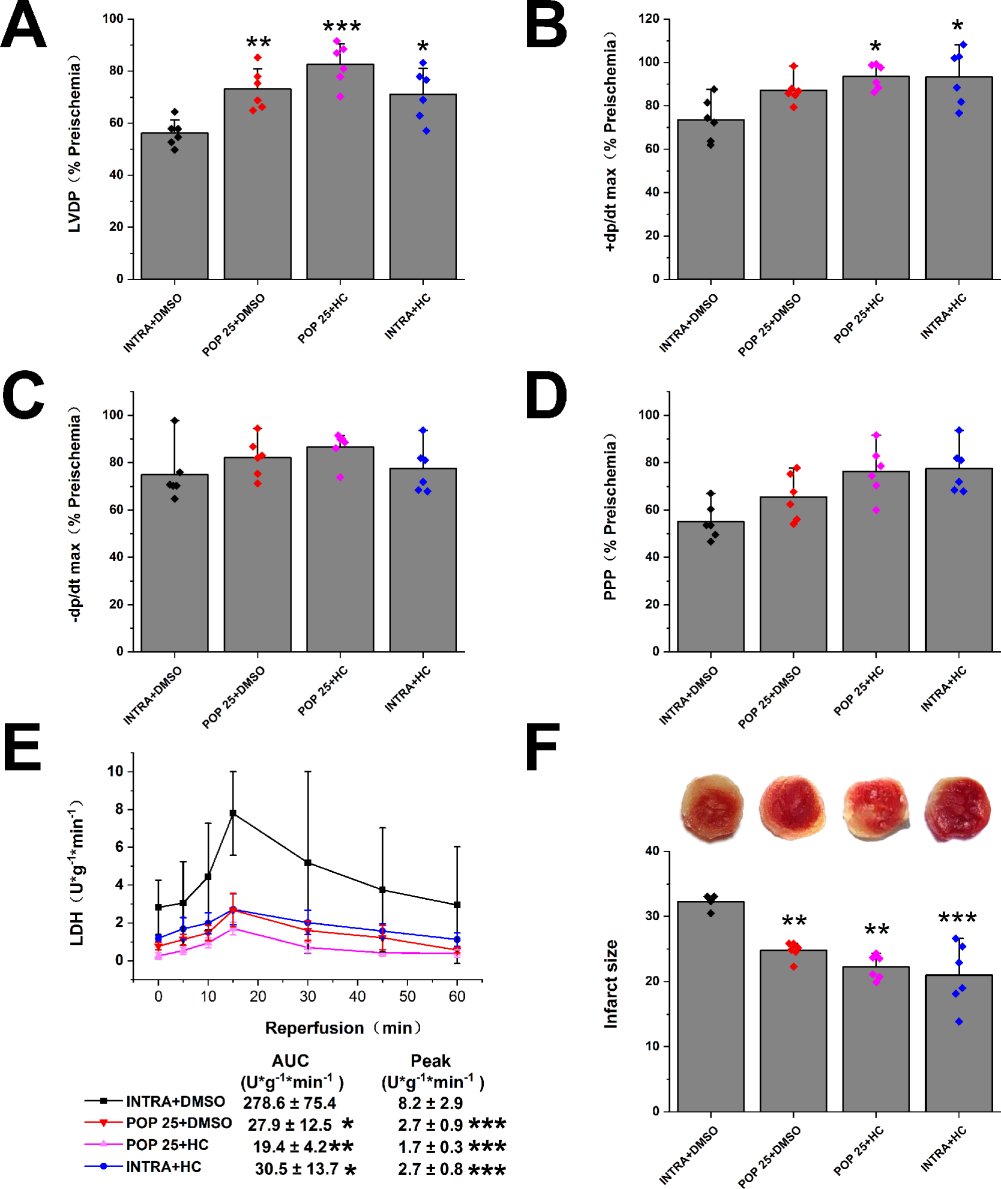
The effect of the combination of propofol and TRPV4 antagonist on myocardial I/R injury. Propofol or intralipid is given 5 minutes before applying TRPV4 antagonist or DMSO. The recovery of LVDP **(A)**, +dP/dt max **(B)**, -dP/dt max **(C)**, and RPP **(D)** 60 minutes after reperfusion displayed as the percentage of respective preischemia values. **(E)** The amount of LDH release in the coronary effluent at different times after reperfusion. **(F)** Representative images of TTC stained LV slices. Infarct size is quantified. Values are presented as mean ± SD, n = 6 for all groups, a one-way ANOVA followed by a Bonferroni test, *p < 0.05, **p < 0.01, ***p < 0.001 *vs.* INTRA + DMSO.

Next, we investigated the effect of propofol on TRPV4 agonist-induced myocardial I/R injury in isolated mice hearts. As shown in **Figure 4** and **Supplementary Figure S4**, compared with INTRA+ DMSO, treatment with TRPV4 agonist GSK1016790A induced the detrimental effects. These effects, however, can be reduced by applying a combination of GSK1016790A and propofol at 50 μM. POP + DMSO had better recovery rates of cardiac function (LVDP, +dp/dt max, and RRR) and less release of LDH as well as infarct size compared with POP + GSK. It appears that propofol (50 μM) cannot completely reverse the detrimental effects induced by GSK1016790A in isolated mice hearts. We further investigated the effect of the combination of propofol and TRPV4 antagonists on myocardial I/R injury in isolated mice hearts. To leave sufficient room for HC-067047 to play an additive protective effect, we reduced the concentration of propofol to 25 μM. As shown in **Figures 5** and **5S**, combining TRPV4 antagonist HC-067047 and propofol produced no additional protective effect compared to propofol or HC-067047 alone. These results suggest that propofol attenuates myocardial I/R injury at least partially *via* the suppression of TRPV4 channel.

### Propofol Attenuates H/R-Induced Intracellular Ca^2+^ Overload and Cell Injury in Cardiomyocytes *Via the* Suppression of TRPV4 Channel

**Figure 6:**
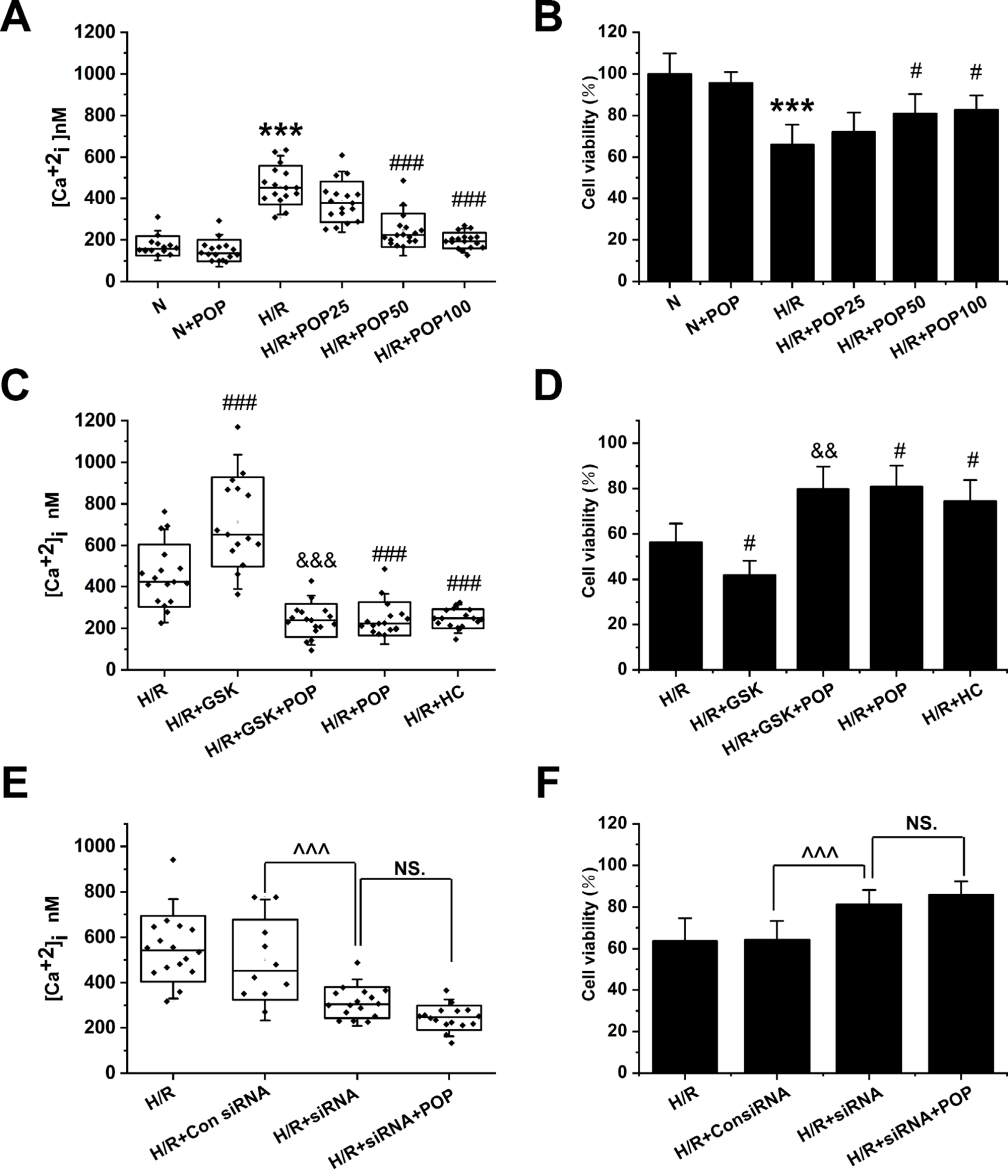
The effect of the combination of propofol and TRPV4 agonist/TRPV4-siRNA on the [Ca^2+^]_i_ and cell viability in H9C2 cells subjected to H/R. Pretreatment with propofol dose-dependently attenuates [Ca^2+^]_i_ (**A**, n = 14 in N group, n = 16 in N + POP and H/R groups, n = 17 in H/R + POP 25, H/R + POP 50, and H/R + POP 100 groups) and increases cell viability (**B**, n = 6 for all groups). (**C**, n = 17 in H/R, H/R + GSK + POP,H/R + POP, H/R + HC, and H/R + HC + POP groups, n = 15 in H/R + GSK group) and (**D**, n = 6 for all groups) show the effects of propofol in the presence of TRPV4 agonist GSK1016790A. (**E**, n = 16 in H/R, H/R + siRNA, and H/R + siRNA + POP groups, n = 10 in H/R + Con siRNA and H/R + POP groups) and (**F**, n = 6 for all groups) show the protective effects of TRPV4-siRNA combined with propofol. Values are presented as mean ± SD, a one-way ANOVA followed by a Bonferroni test, ***p < 0.001 *vs.* N. ^#^p < 0.05, ^###^p < 0.001 *vs.* H/R. ^&&^p < 0.01, ^&&&^p < 0.001 *vs.* H/R+GSK. ^^^p < 0.001 *vs.* HR+Con siRNA, NS. means no statistical significance.

As shown in **Figures 6A, B**, propofol has no obvious effect on the [Ca^2+^]_i_ or cell viability in H9C2 cells under normoxic conditions ([Ca^2+^]_i_: 172.3 ± 47.4 nM *vs.* 149.1 ± 51.2 nM, cell viability: 100 ± 9.8% *vs.* 95.6 ± 5.4%). However, pretreatment with propofol (25, 50 and 100 μΜ) dose-dependently reduced the [Ca^2+^]_i_ (from 464.0 ± 94.1 nM to 383.9 ± 97.8 nM, 245.8 ± 80.8 nM, and 196.9 ± 38.6 nM, respectively) and increased the cell viability in H9C2 cells subjected to H/R (from 65.9 ± 9.6% to 72.0 ± 9.3%, 80.9 ± 9.4%, and 82.6 ± 7.0%, respectively). Moreover, propofol significantly inhibited the enhancement effects of TRPV4 agonist GSK1016790A on H/R-induced intracellular Ca^2+^ overload and cell injury ([Ca^2+^]_i_: 238.0 ± 79.2 nM *vs.* 712.4 ± 216.0 nM, cell viability: 79.8 ± 10.0% *vs.* 41.8 ± 6.3% , **Figures 6C, D**). TRPV4-siRNA protected cells from H/R injury ([Ca^2+^]_i_: 311.9 ± 68.3 nM *vs.* 500.2 ± 178.1 nM, cell viability: 81.4 ± 6.8% *vs.* 64.3 ± 9.0%, **Figures 6E, F**), but this effect was not further enhanced when 50 μΜ propofol was applied to H9C2 cells transfected with TRPV4-siRNA ([Ca^2+^]_i_: 244.1 ± 52.3 nM *vs.* 311.9 ± 68.3 nM, cell viability: 85.9 ± 6.4% *vs.* 81.4 ± 6.8%). This indicates that propofol has no further cardioprotective effect in H9C2 cells transfected with TRPV4-siRNA. Therefore, we could infer that propofol attenuates H/R-induced intracellular Ca^2+^ overload and cell injury in H9C2 cells at least partially *via* the suppression of TRPV4 channel.

**Figure 7:**
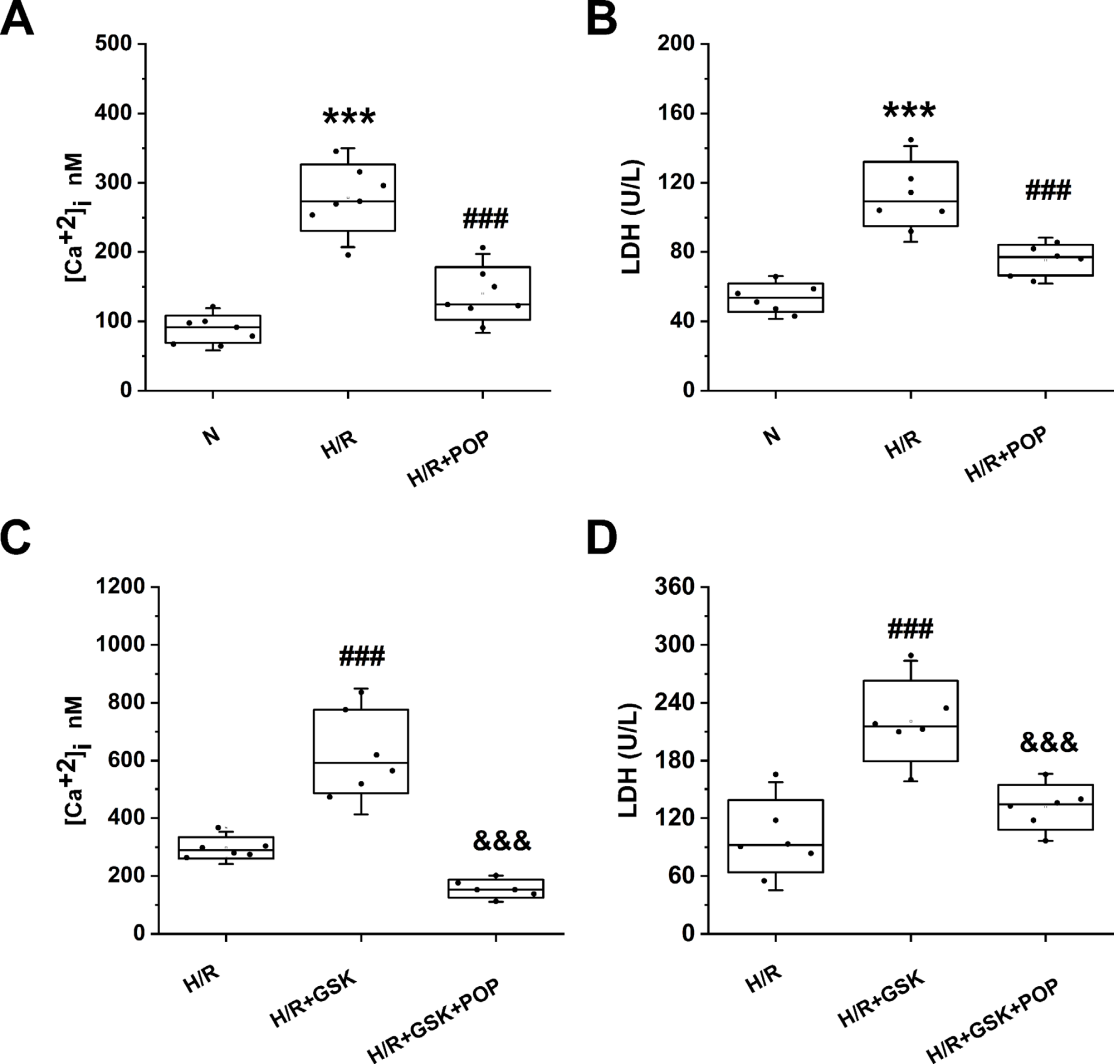
The effect propofol on TRPV4 agonist-induced [Ca^2+^]_i_ and the LDH release in ARVMs subjected to H/R. Pretreatment with 50 µM propofol attenuates [Ca^2+^]_i_ **(A)** and LDH release **(B)**, **(C)** and **(D)** show the effects of propofol in the presence of TRPV4 agonist GSK1016790A. Values are presented as mean ± SD, n = 6 for all groups, a one-way ANOVA followed by a Bonferroni test, ***p < 0.001 *vs.* N. ^###^p< 0.001 *vs.* H/R. ^&&&^p < 0.001 *vs.* H/R+GSK.

We also tested whether propofol had similar effects on ARVMs. ARVMs were isolated from adult rat hearts and were subjected to H/R after 1 day of incubation. **Figure 7** shows H/R-induced [Ca^2+^]_i_ increase (from 88.8 ± 20.0 nM to 278.5 ± 47.8 nM, p < 0.001 *vs.* N) and cell death expressed by the LDH release (from 53.8 ± 8.2 U/L to 113.5 ± 18.5U/L). Pretreatment with 50 μΜ propofol significantly suppressed the above effects ([Ca^2+^]_i_ = 140.4 ± 38.1 nM, LDH = 75.1 ± 8.7 U/L). Similar to the effects in H9C2 cells, propofol reversed the intracellular Ca^2+^ overload and cell death induced by TRPV4 agonist GSK1016790A ([Ca^2+^] _i_: 156.4 ± 30.7 nM *vs.* 630.9 ± 144.6 nM, LDH: 131.4 ± 20.1 U/L *vs.* 220.8 ± 41.7 U/L).

### Propofol Suppresses TRPV4 Channel Function in a Concentration-Dependent Manner in 293T Cells Transfected With hTRPV4

**Figure 8:**
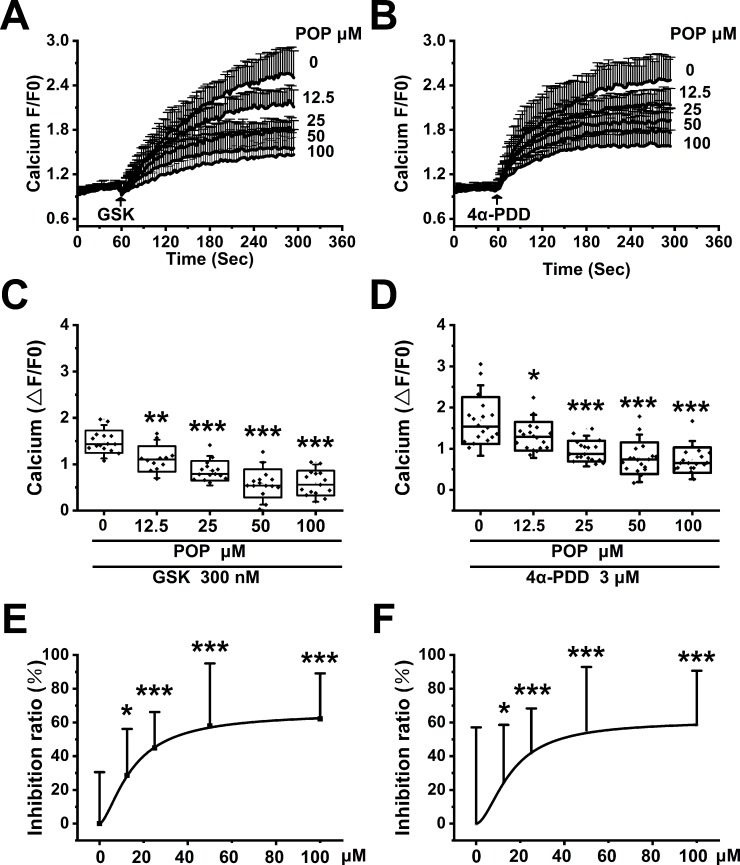
Propofol dose-dependently suppresses the Ca^2+^ influx induced by TRPV4 agonists in HEK-293T cells transfected with hTRPV4. The above graphs show intracellular Ca^2+^ level change induced by 300 nM GSK1016790A **(A)** and 3 μM 4α-PDD **(B)**. Arrows indicate when GSK1016790A and 4α-PDD are applied. Quantitative analyses of the relative changes in Ca^2+^ influx induced by GSK1016790A **(C)** and 4α-PDD **(D)** (∆F/F0). The dose-response curve fitted by the Hill function showing the inhibition of Ca^2+^ influx induced by GSK1016790A **(E)** and 4α-PDD **(F)**. Values are presented as mean ± SD, GSK1016790A: n = 15 in the absence of POP, n = 12 in the presence of propofol at 12.5 µM, n = 15 in the presence of propofol at 25, 50 and 100 µM, 4α-PDD: n = 18 for all groups, a one-way ANOVA followed by a Bonferroni test, *p < 0.05, **p < 0.01, ***p < 0.001 *vs.* without propofol.

To test the hypothesis that propofol inhibits TRPV4 channel function, we measured the Ca^2+^ influx induced by specific TRPV4 agonists GSK1016790A and 4α-PDD in HEK-293T cells transfected with hTRPV4. GSK1016790A and 4α-PDD both induced significant Ca^2+^ influx, which is reduced by pretreatment with propofol (12.5, 25, 50, 100 µM, **Figures 8A, B**). The quantitative analysis of the relative changes (∆F/F0) in Ca^2+^ influx is displayed in **Figures 8 C, D**. The concentration response data fitted by the Hill equation shows a propofol IC_50_ of 39.1 ± 4.5 μΜ under GSK1016790A and 45.3 ± 6.9 μΜ under 4α-PDD (**Figures 8E, F**). Our results proved that propofol suppresses the function of TRPV4 channel in a concentration-dependent manner.

### Propofol Suppresses TRPV4 Channel Function in Cardiomyocytes

**Figure 9:**
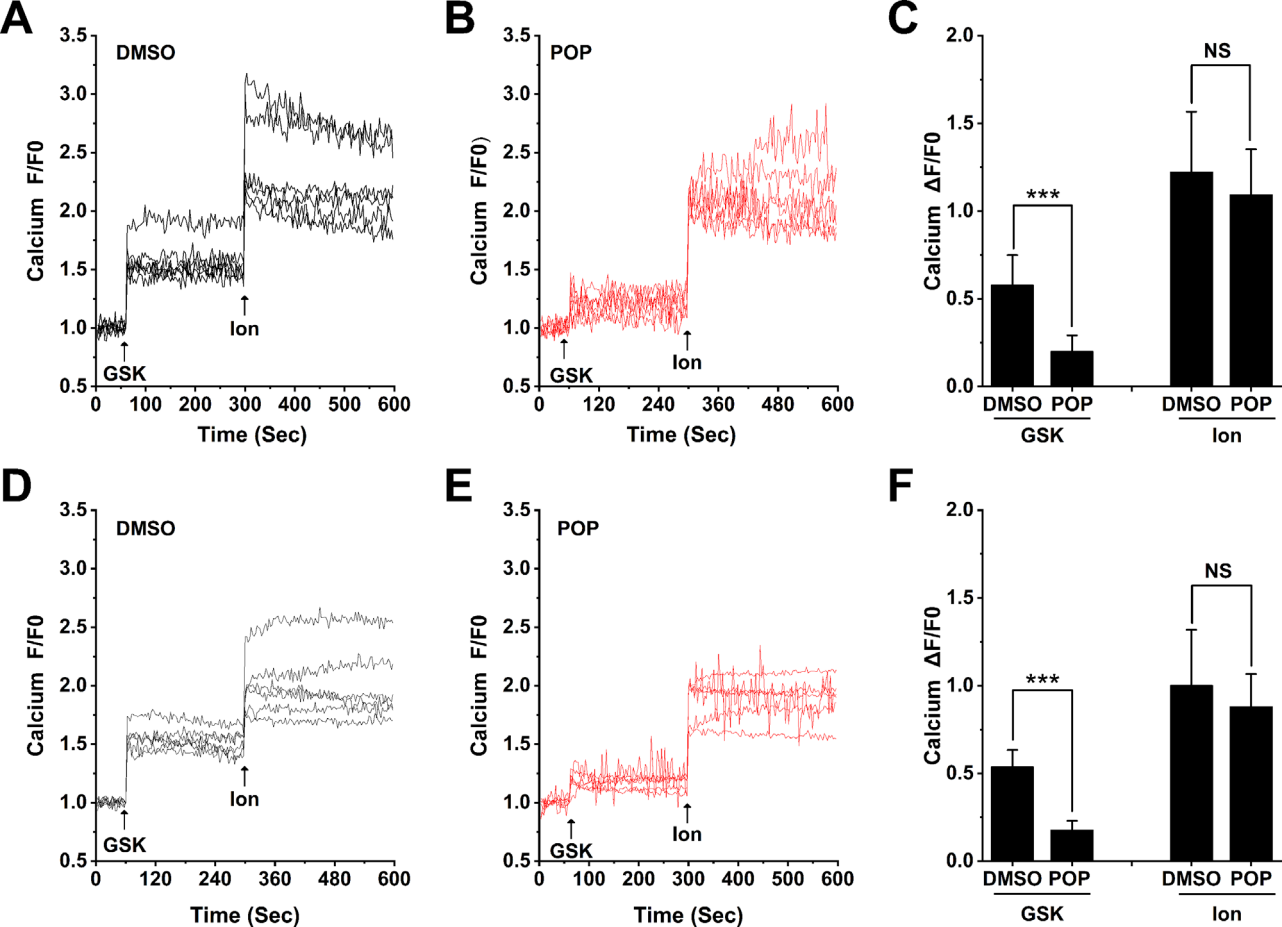
Propofol suppresses the Ca^2+^ influx induced by TRPV4 agonists in cardiomyocytes. Original time-lapse Ca^2+^ influx traces and quantification of GSK1016790A -induced [Ca^2+^]_i_ response in H9C2 cells **(A-C)** and ARVMs **(D-F)**. Arrows indicate when GSK1016790A (300 nM) and Ionomycin (1 μM) are applied. Cells are pretreated with 50 μM propofol for 30 minutes. Ionomycin is set as a positive control. Values are presented as mean ± SD, n = 6 for all groups, a two-tailed t test, ***p < 0.001 *vs.* DMSO, NS. means no statistical significance

Confirming our previous observation (Wu et al., 2017) H9C2 and ARVMs show obvious Ca^2+^ influx after stimulation with 300 nM GSK1016790A (**Figures 9A, D**). Similar to our results from 293T cells transfected with hTRPV4, pretreatment with 50 μM propofol significantly reduced Ca^2+^ influx induced by GSK1016790A, but had no effect on Ca^2+^ influx induced by Ionomycin in H9C2 or ARVMs (**Figures 9 B, E**). The quantitative analysis of the relative changes (∆F/F0) in Ca^2+^ influx in cardiomyocytes is presented in **Figures 9 C, F**, and Ionomycin was set as a positive control. This demonstrates that propofol suppresses the function of TRPV4 channel in cardiomyocytes.

## Discussion

Earlier studies have demonstrated that propofol protects the heart against I/R injury through inhibiting intracellular Ca^2+^ overload (Kim et al., 2008). However, the specific mechanisms are not yet clear. Our previous research has shown that TRPV4 channel functional expression significantly increases in both the *in vivo* and *in vitro* model of myocardial I/R, resulting in intracellular Ca^2+^ overload and myocardial injury (Dong et al., 2017; Wu et al., 2017). This study is the first to prove that propofol attenuates myocardial I/R injury at least partially through the suppression of TRPV4 channel and the subsequent inhibition of intracellular Ca^2+^ overload.

We first confirmed the previous finding that propofol protects isolated hearts from I/R injury in a concentration-dependent manner. However, our results showed that 50 μM propofol had the best protective effects on mice, while Shao et al. and Ko et al. found that 100 μM has the best results on rat (Ko et al., 1997; Shao et al., 2008). The reason might be that the concentration of 100 μM is too high for mice and that high dosage propofol suppresses heart contraction by inhibiting Ca^2+^ influx as well as Ca^2+^-induced excitation-contraction coupling (Li et al., 1997; Han et al., 2016). Therefore, a concentration of 25 or 50 μM was chosen in the following experiment.

Using the Langendorff model, we found that 0.1 μM TRPV4 antagonist HC-067047 significantly promoted the recovery of LVDP, +dP/dt max, -dP/dt max, and RPP, and reduced LDH release as well as infarct size. On the contrary, 20 nM TRPV4 agonist GSK1016790A exacerbated myocardial I/R injury. These results demonstrated that TRPV4 channel activation contributed to myocardial I/R injury in *ex vivo* isolated mice hearts, which is consistent with the results of our previous *in vivo* and *in vitro* studies (Dong et al., 2017; Wu et al., 2017). We further investigated the effects of the combination propofol and TRPV4 agonist/antagonist on myocardial I/R injury. Our results show that the detrimental effects induced by GSK1016790A were reversed by pretreatment with propofol *in vitro* but not in *ex vivo* isolated hearts. TRPV4 activation during I/R is likely mediated by an endogenous mechanism that is less vigorous than GSK1067790A. Moreover, the exogenous GSK1067790A and the endogenous TRPV4 activators, e.g. EETs, may activate TRPV4 *via* different mechanisms. TRPV4 activation by I/R alone could be reduced by propofol. Furthermore, the combination of propofol and TRPV4 antagonist HC-067047 did not create additional protective effects *ex vivo*. Similar results were also found in H9C2 transfected with TRPV4-siRNA. This suggested that propofol reduced myocardial I/R injury at least partially *via* the suppression of TRPV4 channel.

Ca^2+^ is vital for heart contraction. However, after myocardial I/R, intracellular Ca^2+^ overload impairs left ventricular mechanical function (Steenbergen et al., 1993; Meissner and Morgan, 1995). A number of pathways have been found to mediate cardiomyocytes Ca^2+^ overload, including the Na^+^/Ca^2+^ exchanger, Ca^2+^-ATPase, L-type Ca^2+^ channel, sarcoplasmic reticulum Ca^2+^ release channel, and TRPV4 channel (Takahashi et al., 1994; Garcia-Dorado et al., 2012; Wu et al., 2017; Jones et al., 2019). One of our previous studies shows that the activation of TRPV4 channel contributes to H/R-induced intracellular Ca^2+^ overload in cardiomyocytes (Wu et al., 2017). In this study, we also measured the [Ca^2+^] _i_ in H9C2 cells and ARVMs subjected to H/R. Pretreatment with propofol inhibited H/R-induced [Ca^2+^]_i_ increase and also prevented the effect of TRPV4 agonist GSK1016790A on [Ca^2+^]_i_. Furthermore, we found no obvious difference in [Ca^2+^]_i_ between H/R + POP, H/R + TRPV4 siRNA, and H/R + TRPV4 siRNA + POP groups. Therefore, we assume that propofol attenuates H/R-induced intracellular Ca^2+^ overload through the suppression of TRPV4 channel. In addition, many previous studies have already demonstrated that the blockage of TRPV4 channel is a promising target for I/R therapy (Jie et al., 2015; Jie et al., 2016; Dong et al., 2017; Jones et al., 2019; Wu et al., 2019).

In order to investigate whether propofol could inhibit TRPV4-mediared effects, we recorded the Ca^2+^ signals in H9C2 cells, ARVMs, and HEK-293T cells transfected with hTRPV4. Our results showed that propofol suppressed the Ca^2+^ influx induced by TRPV4 agonists GSK1016790A and 4α-PDD in a concentration-dependent manner. The IC_50_ is 39.1 ± 4.5 μΜ and 45.3 ± 6.9 μΜ, respectively, both of which were close to the concentration levels of 35 μΜ in clinical applications (Mathur et al., 1999), indicating that propofol can suppress TRPV4 channel to induce cardioprotective effects under I/R.

Propofol has also been reported to reduce Ca^2+^ entry *via* TRPC5 (Bahnasi et al., 2008) with an estimated IC_50_ value of 84.5 μΜ in transfected HEK293 cells. TRPC5 is present in the heart and is upregulated in the cardiac myocyte hypertrophy model and human heart failure conditions (Lau et al., 2016). Therefore, more research is required to determine whether propofol-induced cardiac protection acts through a TRPC5-dependent mechanism. Propofol, as a commonly used anesthetic in clinical practice, is recognized as a safe and effective drug. However, unwanted side effects with propofol use have been documented and include hypotension, postoperative pain, and propofol-related infusion syndrome, a rare but seriously life-threatening complication (Mirrakhimov et al., 2015). Activation of TRPA1 and TRPV1 has been demonstrated to contribute to propofol-induced vasodilation and pain (Nishimoto et al., 2015; Sinharoy et al., 2017). Interestingly, the concentrations of propofol that induced Ca^2+^ influx *via* TRPA1 (EC_50_ = 65.4 μM) and TRPV1 (90 μM) are similar to those that affect TRPV4. Moreover, TRPV4 is also abundant in smooth muscle and endothelial cells of blood vessels (Randhawa and Jaggi, 2015). TRPV4 activation involves in shear stress-induced vasodilation as well as the damage of the vascular barrier. Whether TRPV4 inhibition involved in propofol-induced vasoreactivity or propofol-related infusion syndrome requires further research.

Our study has a few limitations. First, *ex vivo* or *in vitro* findings might be different from *in vivo* results. Second, although we discovered that propofol suppresses TRPV4 channel, which has yielded new insights into how propofol induces cardioprotective effects, the binding sites of propofol and TRPV4 channel are still unclear. This requires further research that applies site-directed mutagenesis methods, gene delivery techniques, and advanced computational technologies. In addition, although common in other studies, the knockdown efficiency (50%) of TRPV4-siRNA in the present study is low (Adapala et al., 2013; Suresh et al., 2015).

In conclusion, we conducted a series of *ex vivo* and *in vitro* experiments and demonstrated that propofol directly inhibited Ca^2+^ entry *via* TRPV4 channel, which is a newfound mechanism whereby propofol reduces intracellular Ca^2+^ overload and subsequently attenuates myocardial I/R injury. Due to the widespread expression and numerous effects of TRPV4 channel, the findings of this research are also relevant to other organ systems, including the respiratory, digestive, and urinary systems (Randhawa and Jaggi, 2015). Recent evidence has demonstrated that TRPV4 channel activation is related to pulmonary edema, gastrointestinal disorders, and bladder dysfunction. Moreover, a recent review article has highlighted the importance of TRPV4 channel in the pathogenesis of various diseases (White et al., 2016). Therefore, we think the use of propofol during the perioperative period is potentially beneficial to the prevention of TRPV4 channel-related diseases. However, this is beyond the scope of this study and requires further research. In addition, future research will need to examine whether other general anesthetics, such as volatile anesthetics or etomidate, also suppress TRPV4 channel to produce cardioprotective effects.

## Data Availability Statement

The raw data supporting the conclusions of this manuscript will be made available by the authors, without undue reservation, to any qualified researcher.

## Ethics Statement

All procedures concerning animal use were performed in adherence to the National Institutes of Health Guide for the Care and Use of Laboratory Animals (NIH Publications No. 8023, revised 1978) and approved by Tongji Medical College Committee on Animal Care.

## Author Contributions

BW, QW, NZ, ZH, KG, and YD contributed conception and design of the study; BW, QW, JL, SZ, HL, CY, QD, and NZ organized the database; BW, QW, and JL performed the statistical analysis; BW, QW, and JL wrote the first draft of the manuscript; ZH, KG, and YD wrote sections of the manuscript. All authors contributed to manuscript revision, read and approved the submitted version.

## Funding

This research was funded by the National Nature Science Foundation of China, grant number 81470421 (YD), 81770328 (YD), and 81700332 (NZ).

## Conflict of Interest

The authors declare that the research was conducted in the absence of any commercial or financial relationships that could be construed as a potential conflict of interest.
