# Supplementary material for: Propofol Induces Cardioprotection Against Ischemia-Reperfusion Injury via Suppression of Transient Receptor Potential Vanilloid 4 Channel
Source: Front Pharmacol. 2019 Oct 4;10:1150. doi: 10.3389/fphar.2019.01150 (PMC6788301; doi:10.3389/fphar.2019.01150)
Supplement: Supplementary file 1 [file Table_1.docx]

Supplementary Material

# Materials and Methods

## Reverse transcription (RT)-PCR amplification and Quantitative (q) PCR

Total RNAs were extracted from cultured H9C2 cells. Primers used were as follows: TRPV4: forward, 5′-CCCGAGAGAACACCAAGTTTG-3′, reverse, 5′-GACCGTCA- TTGTTAAGCACAGTCT-3′; Actin: forward, 5′-CGTTGACATCCGTAAAGACC-3′, reverse, 5′-TAGAGCCACCAATCCACACA-3′. The thermal cycler program used for real-time quantitative PCR amplification included a denaturation step (95°C for 30 s), an annealing step (60°C for 30 s), and a primer extension step (72 °C for 30 s, 39 cycles). Amplified products were separated in 2% agarose gel by gel-electrophoresis and stained with ethidium bromide. We calculated the relative expression quantity 2-^ΔΔCt^ value of TRPV4 to compare the differences between Con siRNA and TRPV4-siRNA groups.

## Western Blot

Total protein was extracted from heart tissues and cultured H9C2 cells. Each sample had an equal amount of protein (20 μg), which was separated by 10% SDS-PAGE gel electrophoresis and transferred onto PVDF membranes. The membranes were blocked using nonfat milk and then incubated in primary antibodies, rabbit anti-TRPV4 (Alomone labs, Jerusalem, Israel), and rabbit anti-β-actin (Biossci, Wuhan, China) at 4 °C overnight. The membranes were subsequently incubated in horseradish peroxidase-conjugated secondary antibodies, goat anti-rabbit immunoglobulin G (Biossci, Wuhan, China), and anti-biotin (Cell Signaling Technology, Danvers, MA, USA) for 2 h at room temperature. Immunoreactivity was detected by Bio-Rad ChemiDoc XRS (Bio-Rad, Hercules, CA, USA) and quantified by Image Lab™ Software.





**Supplemental Figure 1** Specific small interfering RNA (siRNA) modulates TRPV4 channel function in H9C2 cells. Relative TRPV4 mRNA and protein levels were detected by RT-PCR (A), qRT-PCR (B), and Western blot (C and D). Graph (E) and a quantitative analysis (F) show that the relative changes in intracellular Ca2+ influx induced by 300 nM GSK in H9C2 cells transfected with TRPV4-siRNA. Arrows indicate when GSK was applied. Values are presented as mean ± SD, n = 6 to 7 for all groups. *p < 0.05, ***p < 0.001 vs. Con siRNA.

**

**

**Supplemental Figure 2** The representative traces of left ventricular pressure (left) and rate of pressure change (dP/dt, right) in hearts perfused Intra (A), POP 25 (B), POP 50 (C), and POP 100 (D) during ischemia, and following reperfusion.





**Supplemental Figure 3** The representative traces of left ventricular pressure (left) and rate of pressure change (dP/dt, right) in hearts perfused DMSO (A), GSK (B), and HC (C) during ischemia, and following reperfusion.





**Supplemental Figure 4** The representative traces of left ventricular pressure (left) and rate of pressure change (dP/dt, right) in hearts perfused INTRA+DMSO (A), INTRA+GSK (B), POP 50+GSK (C), and POP 50+DMSO (D) during ischemia, and following reperfusion.





**Supplemental Figure 5** The representative traces of left ventricular pressure (left) and rate of pressure change (dP/dt, right) in hearts perfused INTRA+DMSO (A), POP 25+DMSO (B), POP 25+HC (C), and INTRA+HC (D) during ischemia, and following reperfusion.

**Supplemental** **Table 1：** **HR, LVDP, +dp/dt max, -dp/dt max, RPP after 15 min of equilibration**

|  | HR | LVDP | +dp/dt max | -dp/dt max | RPP |
| --- | --- | --- | --- | --- | --- |
|  | (beats/min) | (mmHg) | (mmHg/s) | (mmHg/s) | (mmHg*beats*min^-1^) |
| INTRA | 389 ± 31 | 94.9 ± 13.0 | 4280.3 ± 438.2 | -3438.1 ± 318.2 | 36899.2 ± 5712.9 |
| POP 25 | 393 ± 54 | 98.1 ± 16.9 | 4856.5 ± 670.9 | -3754.1 ± 600.4 | 38696.0 ± 8634.0 |
| POP 50 | 388 ± 11 | 92.5 ± 14.9 | 4819.2 ± 598.9 | -3606.7 ± 307.7 | 35789.1 ± 5167.4 |
| POP 100 | 397 ± 43 | 101.9 ± 11.8 | 5189.3 ± 595.7 | -3925.3 ± 470.3 | 40328.3 ± 5187.0 |
| DMSO | 354 ± 13 | 98.9 ± 10.5 | 4494.4 ± 286.1 | -3470.7 ± 140.4 | 34885.9 ± 3004.8 |
| GSK | 355 ± 18 | 99.7 ± 16.9 | 4359.7 ± 545.5 | -3644.3 ± 384.1 | 35141.3 ± 4542.3 |
| WT+HC | 375 ± 28 | 89.6 ± 11.8 | 4263.2 ± 463.2 | -3421.9 ± 265.8 | 33387.7 ± 2986.4 |
| INTRA+DMSO | 356 ± 20 | 89.2 ± 13.5 | 5460.7 ± 851.4 | -4177.0 ± 533.9 | 31725.8 ± 5023.5 |
| INTRA+GSK | 363 ± 34 | 106.3 ± 24.7 | 5838.9 ± 739.0 | -4423.9 ± 373.0 | 38271.7 ± 7591.0 |
| POP 50+GSK | 358 ± 61 | 94.4 ± 13.1 | 4981.6 ± 589.4 | -3805.2 ± 415.9 | 33746.0 ± 6898.0 |
| POP 50+DMSO | 375 ± 62 | 86.5 ± 13.5 | 4935.6 ± 618.9 | -3677.2 ± 520.1 | 32263.8 ± 6473.6 |
| POP 50+HC | 357 ± 26 | 87.8 ± 7.5 | 4698.7 ± 410.4 | -3561.1 ± 501.1 | 31372.5 ± 3630.4 |
| POP 25 + DMSO | 336 ± 62 | 80.9 ± 9.1 | 5741.0 ± 1008.2 | -4894.6 ± 992.1 | 29104.6 ± 4275.8 |
| POP 25 + HC | 349 ± 52 | 85.7 ± 5.6 | 5658.1 ± 751.8 | -5070.1 ± 608.9 | 30057.4 ± 5513.7 |
| INTRA + HC | 429 ± 55 | 87.8 ± 7.5 | 5979.7 ± 304.9 | -5470.1 ± 394.3 | 35450.2 ± 4665.2 |

Values are presented as mean ± SD, n = 6 for all groups. H R: heart rate; LVDP: left ventricular developed pressure; +dp/dt max: maximum rate of rise of the left ventricular contraction velocity; -dp/dt max: maximum rate of rise of the left ventricular relaxation velocity; RPP: rate pressure product (RPP = LVDP*HR).

**Supplemental** **Table 2： HR, LVDP, +dp/dt max, -dp/dt max, RPP before ischemia**

|  | HR | LVDP | +dp/dt max | -dp/dt max | RPP |
| --- | --- | --- | --- | --- | --- |
|  | (beats/min) | (mmHg) | (mmHg/s) | (mmHg/s) | (mmHg*beats*min^-1^) |
| INTRA | 370 ± 43 | 95.2 ± 19.6 | 4238.9±638.1 | -3423.5 ± 446.8 | 35510.4 ± 9523.6 |
| POP 25 | 350 ± 68 | 106.7 ± 11.0 | 5038.4 ± 531.0 | -3762.9 ± 312.8 | 37153.3 ± 7514.3 |
| POP 50 | 346 ± 20 | 98.1 ± 11.0 | 5001.3 ± 589.1 | -3677.9 ± 130.1 | 33740.8 ± 2991.1 |
| POP 100 | 297 ± 42 | 110.9 ± 12.5 | 5179.5 ± 344.2 | -3900.5 ± 290.0 | 32899.2 ± 3999.5 |
| DMSO | 349 ± 14 | 94.4 ± 7.1 | 4492.0 ± 510.7 | -3390.7 ± 321.4 | 32980.3 ± 4676.3 |
| GSK | 367 ± 51 | 101.9 ± 11.0 | 4858.7 ± 647.9 | -3806.1 ± 292.7 | 37158.9 ± 5267.4 |
| HC | 346 ± 24 | 90.7 ± 10.5 | 4259.2 ± 251.6 | -3414.1 ± 333.9 | 31193.9 ± 3589.2 |
| INTRA+DMSO | 374 ± 36 | 90.6 ± 13.7 | 5994.2 ± 854.0 | -4392.4 ± 578.7 | 33930.8 ± 6074.4 |
| INTRA+GSK | 382 ± 61 | 94.4 ± 16.8 | 5700.2 ± 864.1 | -4320.2 ± 552.6 | 35918.0 ± 6960.1 |
| POP 50+GSK | 332 ± 45 | 91.0 ± 16.4 | 5013.7 ± 647.2 | -3630.7 ± 390.3 | 30290.7 ± 6613.3 |
| WT+POP 50+DMSO | 346 ± 54 | 83.0 ± 15.5 | 4904.1 ± 519.9 | -3559.7 ± 501.7 | 28495.1 ± 5443.6 |
| POP 50+HC | 311 ± 15 | 88.0 ± 14.9 | 4726.8 ± 640.4 | -3561.1 ± 501.1 | 27246.3 ± 7873.8 |
| POP 25 + DMSO | 331 ± 48 | 85.0 ± 9.9 | 5993.6 ± 664.3 | -4633.8 ± 502.0 | 27426.8 ± 3396.1 |
| POP 25 + HC | 318 ± 56 | 84.0 ± 7.0 | 5864.0 ± 620.3 | -5427.6 ± 491.4 | 27830.8 ± 4216.2 |
| INTRA + HC | 425 ± 47 | 79.3 ± 10.4 | 5788.1 ± 695.3 | -5331.6 ± 779.7 | 33413.5 ± 3826.1 |

Values are presented as mean ± SD, n = 6 for all groups. HR: heart rate; LVDP: left ventricular developed pressure; +dp/dt max: maximum rate of rise of the left ventricular contraction velocity; -dp/dt max: maximum rate of rise of the left ventricular relaxation velocity; RPP: rate pressure product (RPP = LVDP*HR).
